# Real-world Settings for the Surgical Treatment of Neurofibroma in Patients with Neurofibromatosis Type 1

**DOI:** 10.31662/jmaj.2023-0161

**Published:** 2024-02-05

**Authors:** Mayumi Ota, Yoshimasa Nobeyama, Akihiko Asahina

**Affiliations:** 1 Department of Dermatology, Jikei University School of Medicine, Tokyo, Japan

**Keywords:** neurofibromatosis type 1, neurofibroma, surgery, tumor burden, COVID-19 pandemic, medical access

## Abstract

**Introduction:**

Even though an MEK inhibitor has been recently launched, neurofibroma still negatively affects the well-being of patients with neurofibromatosis type 1 (NF1). The coronavirus disease 2019 (COVID-19) pandemic resulted in restricted access to medical care. The present study was conducted to investigate the real-world settings of patients with NF1 who underwent surgery with or without restricted medical access during the COVID-19 pandemic.

**Methods:**

Based on data obtained from medical records, the present study examined 123 and 260 patients who underwent surgery for neurofibromas with and without restricted medical access, respectively.

**Results:**

The mean numbers of surgeries performed during the periods with and without restricted medical access were 5.8 and 9.8 per month, respectively, and there were 1.18- and 1.46-fold more female patients than male patients for each group, respectively. Regardless of whether medical access was restricted, the majority of patients who underwent surgery were middle-aged females with multiple or severe neurofibromas and mild extracutaneous symptoms. Tumor burden was the most common reason for surgery. However, cutaneous neurofibromas were more likely to be treated than plexiform neurofibromas under restricted medical access.

**Conclusions:**

Patients with NF1, particularly middle-aged females with severe cutaneous manifestations and mild extracutaneous manifestations, still underwent surgery for neurofibromas regardless of whether medical access was restricted.

## Introduction

Neurofibromatosis type 1 (NF1), or von Recklinghausen’s disease, is an autosomal-dominant neurocutaneous syndrome that occurs in approximately 1/3000 births with no sex difference ^(1), (2)^. NF1 is caused by mutations in the *NF1* gene that encodes neurofibromin, which suppresses the rat sarcoma viral oncogene homolog /mitogen-activated protein kinase pathway. NF1 presents with various symptoms, including cutaneous, nodular plexiform, and diffuse plexiform neurofibromas as well as malignant peripheral nerve sheath tumor, large pigmented macules, café-au-lait macules, axillary freckling, glioma, scoliosis, iris nodules, and impaired intelligence. Neurofibroma is one of the most common symptoms in patients with NF1. It develops during childhood and increases in size and number with aging, affecting 99% of patients with NF1. Its manifestation harms the quality of life of patients, affecting their mood and general daily and social lives ^(3), (4)^.

An open-label, multicenter, single-arm, phase II SPRINT trial on selumetinib that studied 50 symptomatic children with inoperative plexiform neurofibroma revealed that 68% achieved a partial response and that the estimated 3-year progression-free survival rate was 84% ^(5)^. The SPRINT trial also reported clinical improvements in patient- and parent-reported assessments of pain, range of motion, disfigurement, and quality of life. Based on these findings, selumetinib was recently approved for the treatment of inoperative plexiform neurofibroma in children with NF1. However, information on the effects of selumetinib is not complete and, as of 2023, it is still not available for all types of neurofibroma or all patients with NF1. Therefore, surgery remains an important option for many patients with NF1 with neurofibroma.

Chamseddin et al. reported that surgery for cutaneous neurofibroma significantly improved the quality of life as measured by the Dermatology Life Quality Index ^(6)^. Guiraud et al. examined 170 patients with NF1 (60 male patients and 110 female patients; mean age of 39 years) in four European countries ^(3)^. The majority of patients were treated surgically; 111 (65%) and 64 (38%) patients underwent surgical excision and laser treatments, respectively, showing that surgery is still the main therapeutic option for neurofibroma in many patients with NF1.

Coronavirus disease 2019 (COVID-19) is a respiratory infectious disease caused by a new severe acute respiratory syndrome coronavirus-2 ^(7)^. The COVID-19 pandemic started in 2020 and infected more than 600 million individuals by the end of 2022 ^(8)^. Medical access, particularly for the treatment of benign and non-emergency diseases, such as the surgical treatment of neurofibroma in patients with NF1, was restricted during the emergency and semi-emergency states of COVID-19 measures in Japan.

Information on patients with NF1 who undergo surgery is currently limited. Therefore, the present study was conducted to obtain a more detailed understanding of real-world settings for the surgical treatment of neurofibroma in patients with NF1 with or without restricted medical access during the COVID-19 pandemic.

## Materials and Methods

### Patients

The Ethics Committee of the Jikei University School of Medicine approved the study protocol (approval code: 32-073), and all patients provided informed consent with an opt-out form. Between January 2018 and December 31, 2021, we recruited Japanese patients with NF1 who met the following criteria (Supplementary Table 1): i) referral to the Department of Dermatology, Jikei University School of Medicine; ii) fulfillment of the revised diagnostic criteria described by Legius et al. in 2021 ^(9)^; and iii) underwent surgery for neurofibroma (surgical excision of neurofibroma) as requested.

### Assessments

The following information was obtained from the patient’s medical records: age, sex, the number of excised neurofibromas, the type of excised neurofibroma, the reasons for undergoing surgery (cosmetic disturbance, pain, itch, and tumor burden), and the DNB severity classification, which is a severity scale to evaluate dermatological, neurological, and bone manifestations ^(10)^. The term “tumor burden” was defined as the unpleasant feeling that the volume of neurofibromas is excessive despite the absence of unpleasant sensations or cosmetic disturbances.

The clinical definitions of the types of neurofibromas are as follows: Cutaneous neurofibroma is a neurofibroma that presents a protrusive soft nodule on the skin. Nodular plexiform neurofibroma is a neurofibroma that presents with a subcutaneous nodule, which is commonly aggregated into the moniliform pattern. Diffuse plexiform neurofibroma is a neurofibroma that presents with a large soft mass, which involves various tissues, including skin, fat, muscle, bone, and nerves. A histopathological examination was performed to confirm if an excised lesion was a neurofibroma.

The D-classification is defined as follows: D1, pigmented macules, and a few neurofibromas; D2, pigmented macules and many neurofibromas; D3, numerous neurofibromas (>1000 in number, >1cm in size); D4, severe plexiform neurofibromas or a malignant peripheral nerve sheath tumor. The N-classification is defined as follows: N0, no neurological symptoms; N1, either or both neurological symptoms (e.g., paralysis or pain) and abnormal neurological findings; N2, severe or progressive neurological symptoms. The B-classification is defined as follows: B0, no bone lesions; B1, a mild or moderate bone lesion (deformity in the spine or extremities that does not require treatment); B2, a severe bone lesion (dystrophic type or spine deformity that requires surgery (e.g., scoliosis or kyphosis)), severe bone deformity in the extremities (e.g., pseudarthrosis and fracture), or a defect of the skull or facial bone.

The declaration of emergency and semi-emergency states due to the COVID-19 pandemic was sometimes lifted between April 2020 and December 2021 by the Japanese government. During this time, our institute restricted hospital admission and general anesthesia and demanded that we cancel or postpone elective surgery if the patient accepted it. We then define these 21 months as a restricted medical access period. On the other hand, our institute and government did not restrict clinical affairs in the 27 months between January 2018 and March 2020. These 27 months were defined as a normal medical access period.

### Statistical analysis

Statistical analyses were performed using SPSS version 22 software (IBM, Armonk, NY). The Mann-Whitney U test and Pearson’s chi-squared test were conducted to analyze quantitative and qualitative differences, respectively. Multiple correspondence analysis was employed to elucidate the relationships between the number of excised neurofibromas, the DNB classifications, and the reasons to undergo surgery. *P* ≤ 0.05 was considered to be significant.

## Results

**Figure 1.**
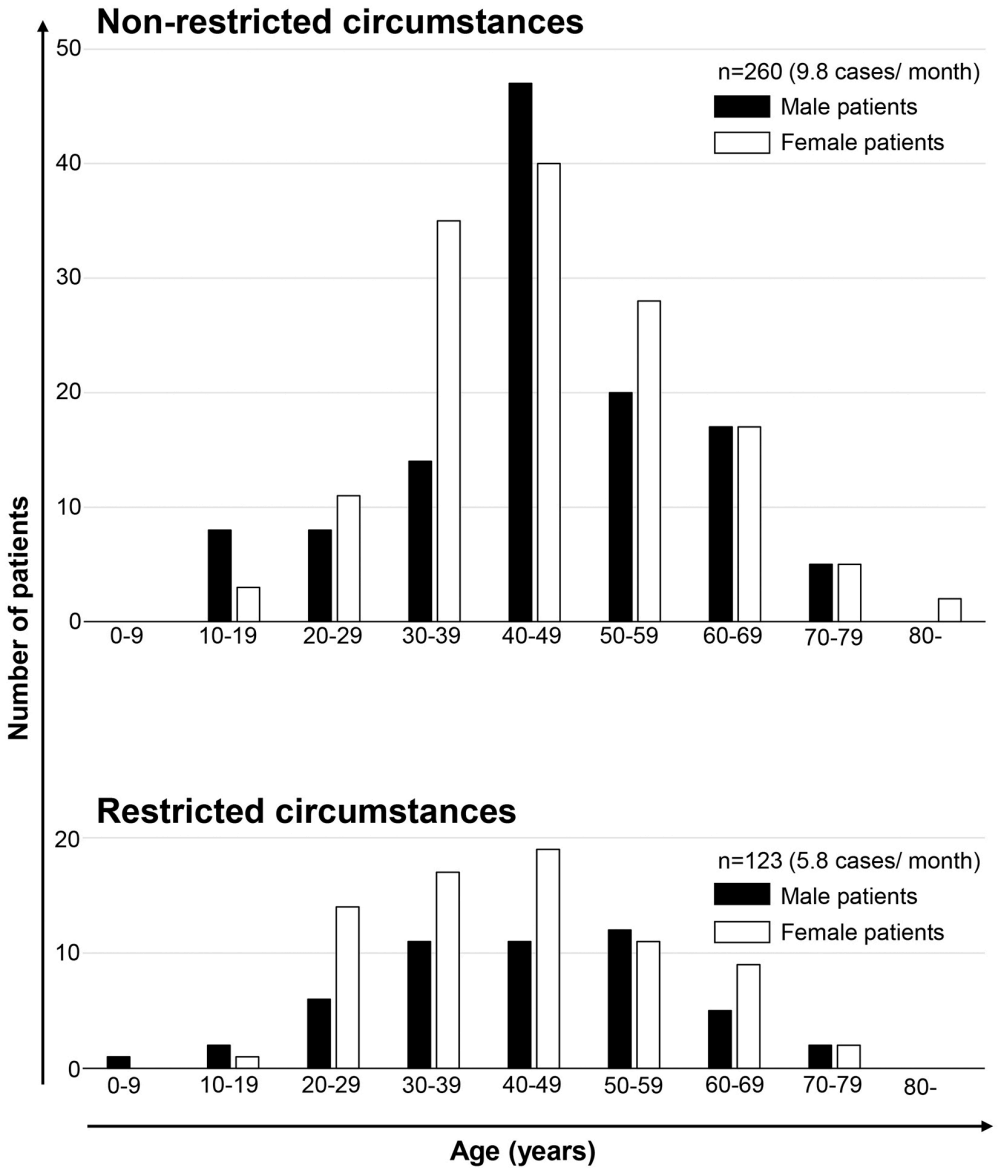
Age and sex distribution in patients who underwent surgery for neurofibroma The upper and lower graphs illustrate data under normal and restricted medical access, respectively. The vertical and horizontal axes indicate the number of patients and age (years), respectively. The black and white bars indicate male and female patients, respectively.

In total, 383 patients with NF1 underwent surgery for neurofibroma at our department between January 1, 2018, and December 31, 2021. The number of surgeries performed was 260 in the normal access period (mean: 9.8 per month) and 123 in the restricted medical access period (mean: 5.8 per month). The mean ages of patients in the normal medical access and restricted medical access periods were 46.3 and 43.7 years old, respectively, with no significant difference. There were 1.18- and 1.46-fold more female patients than male patients during the normal medical access and restricted medical access periods, respectively (Table 1 and Figure 1).

**Table 1.** Comparison between Patients with NF1 with and without Restricted Medical Access.

Parameters	Non-restricted	Restricted	*p*-value
Mean age ± standard deviation (years)	46.3 ± 14.0	43.7 ± 14.3	0.065
Sex distribution			0.346
Male	119	50
Female	141	73
D-classification in each patient			<0.001*
D1	3	5
D2	62	58
D3	130	38
D4	65	22
N-classification in each patient			0.855
N0	175	86
N1	77	34
N2	8	3
B-classification in each patient^ǂ^			0.440
B0	167	81
B1	68	34
B2	25	7
Type of excised neurofibroma in each patient
Cutaneous neurofibroma	230/260 (88.5%)	120/123 (97.6%)	0.003*
Nodular plexiform neurofibroma	21/260 (8.1%)	5/123 (4.1%)	0.145
Diffuse plexiform neurofibroma	23/260 (8.8%)	3/123 (2.4%)	0.020*
Site of excised neurofibroma in each patient
Head/Neck	94/260 (36.2%)	41/123 (33.3%)	0.590
Trunk	146/260 (56.2%)	74/123 (60.2%)	0.459
Arm/Leg	100/260 (38.5%)	44/123 (35.8%)	0.612
Hand/Foot	29/260 (11.2%)	21/123 (17.1%)	0.108
Number of excised neurofibromas in each patient^§^			0.699
1	42	16
2−9	159	79
≥10	58	26
Reason to undergo surgery in each patient (multiple selection allowed)			
Cosmetic disturbance	46/260 (17.7%)	8/123 (6.5%)	0.003*
Pain of tumor	58/260 (22.3%)	15/123 (12.2%)	0.019*
Itch of tumor	2/260 (0.8%)	1/123 (0.8%)	0.964
Burden of tumor	222/260 (85.4%)	112/123 (91.1%)	0.121

^ǂ^Data of Case #40 were excluded to calculate the *p*-value because the data were not available. ^§^Data from Case #47, #70, and #322 were excluded to calculate the *p*-value because the data were not available.

**Figure 2.**
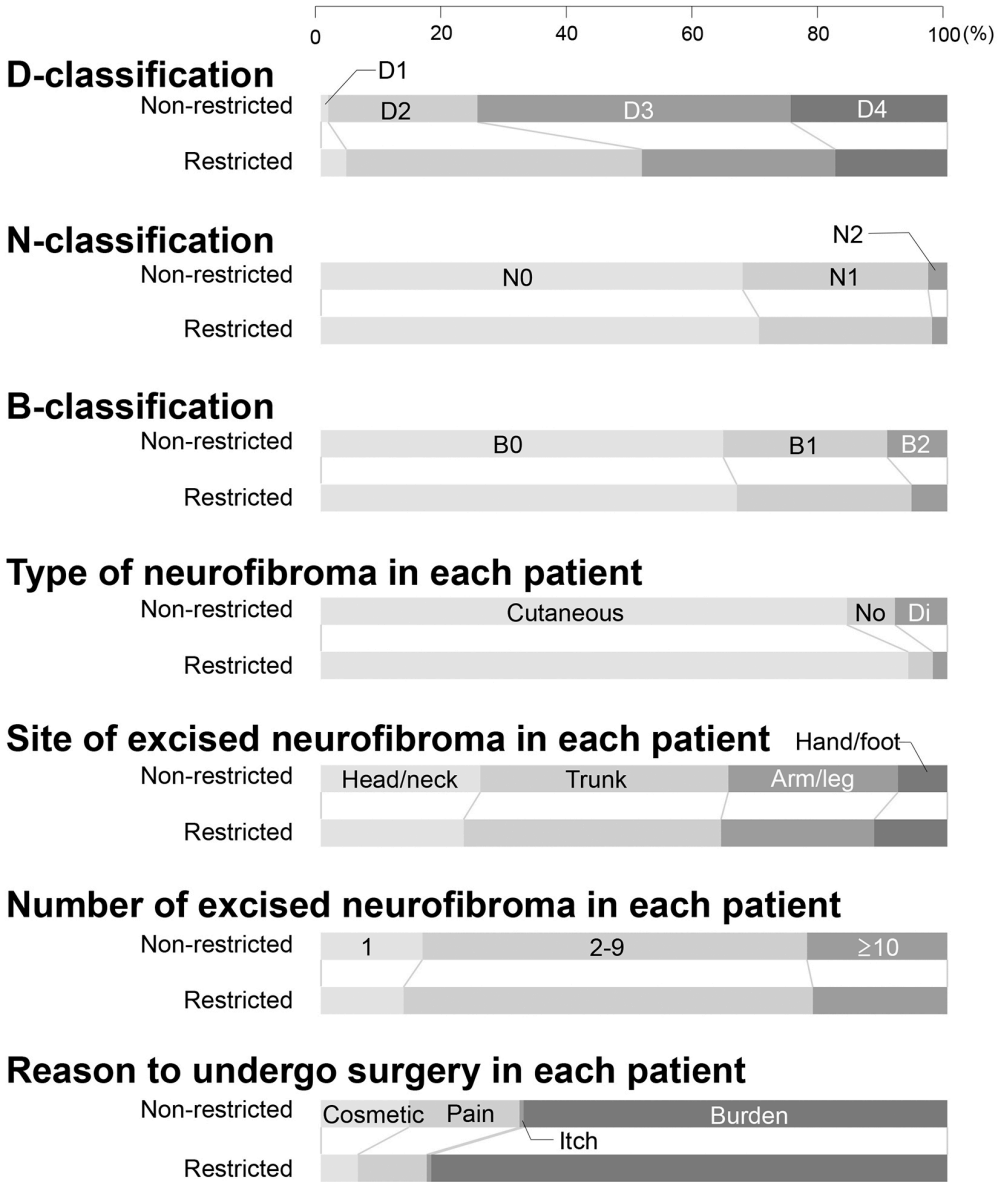
Differences between normal and restricted medical access The differences between normal and restricted medical access are illustrated for the D-, N-, and B-classifications, type of neurofibroma, site of excised neurofibroma, number of excised neurofibroma, and reason to undergo surgery were illustrated. The horizontal axis indicates the percentage. Cutaneous, cutaneous neurofibroma; No, nodular plexiform neurofibroma; Di, diffuse plexiform neurofibroma.

Patients who underwent surgery were classified into D1, D2, D3, and D4 for cutaneous manifestations, N0, N1, and N2 for neurological manifestations, and B0, B1, and B2 for bone manifestations according to the DNB grading system. Many patients who underwent surgery were categorized into N0/N1 and B0/B1 regardless of whether medical access was restricted. On the other hand, many patients who underwent surgery were categorized into D3 during the normal medical access period, or into D2 during the restricted medical access period. The percentages of cutaneous neurofibromas among all types of excised neurofibromas were 97.6% and 88.5% during the restricted medical access and normal medical access periods, respectively, which were significantly different (Table 1 and Figure 2).

No significant differences were observed in the age, sex distribution, the N- and B-classifications, the sites of excised neurofibroma, or the number of excised neurofibromas during both periods.

**Figure 3.**
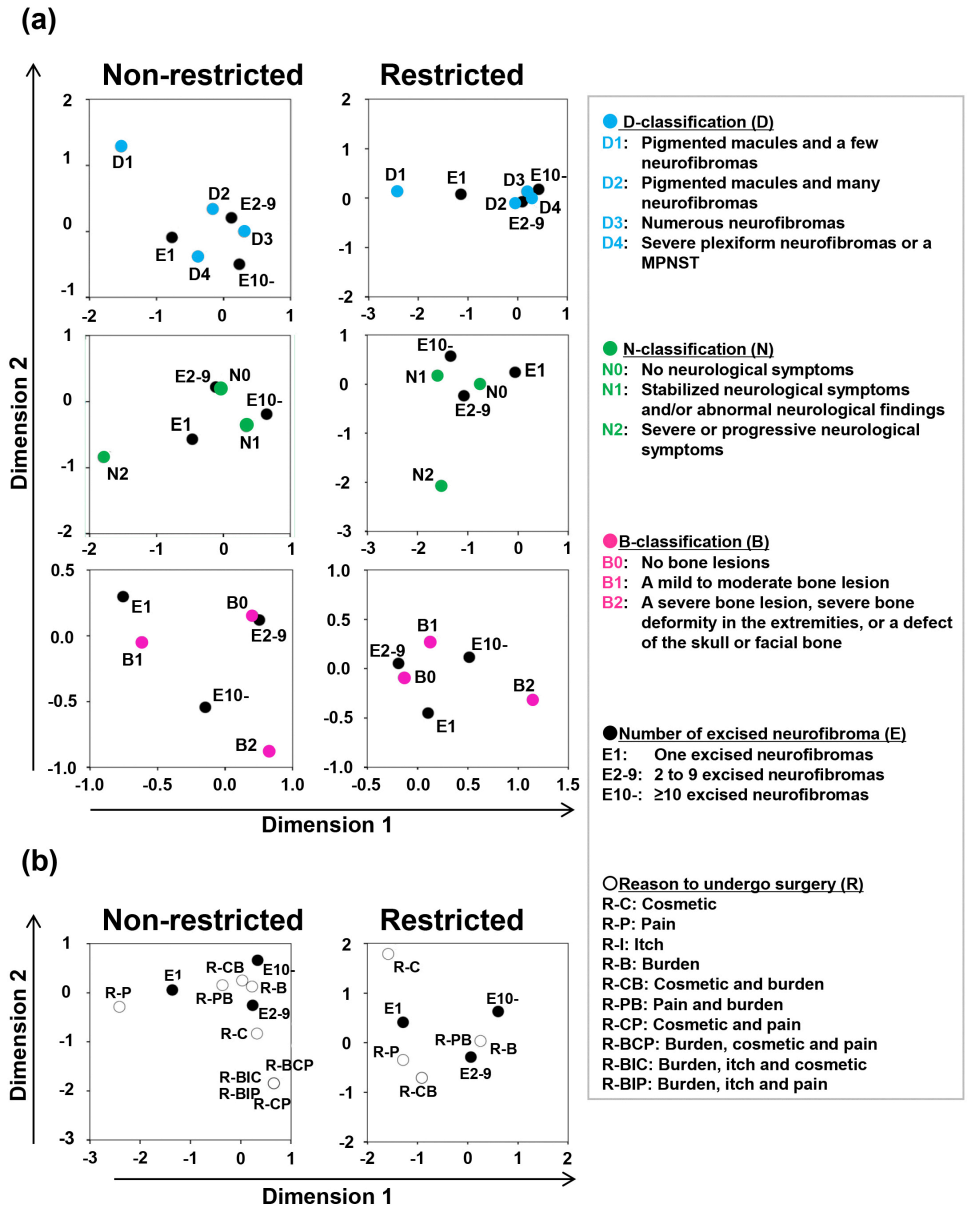
Relationships between the number of excised neurofibromas and severity or reason to undergo surgery Multiple correspondence analyses showed relationships (a) between the number of excised neurofibromas and D-, N-, or B-classification and (b) between the number of excised neurofibromas and reasons to undergo surgery. The vertical and horizontal axes both indicate dimensions. The closed circles indicate the relative positions of patients who underwent surgery for 1 (E1), 2-9 (E2-9), and ≥10 (E10) neurofibromas. The blue, green, and pink closed circles indicate the relative positions of patients with each D-, N-, and B-classification, respectively. Open circles indicate the relative positions of patients with each reason.

The multiple correspondence analysis revealed relationships between the D-, N-, or B-classifications and the number of excised neurofibromas (Figure 3). During the normal medical access period, the patients with one excised neurofibroma had a relatively close relationship with D4, and the patients with 2-9 and ≥10 excised neurofibromas had a relatively close relationship with D2 and D3 as well as with N0 and N1. During the restricted medical access period, the patients with 2-9 and ≥10 excised neurofibromas had a relatively close relationship with D2, D3, and D4, with B0 and B1, and with N0 and N1. Analyses also showed relationships between the reasons to undergo surgery and the number of excised neurofibromas (Figure 3). The patients with 2-9 and ≥10 excised neurofibromas had a relatively close relationship with the tumor burden as the reason to undergo surgery in both periods.

The relationships between the neurofibroma subtypes and the reasons to undergo surgery for neurofibroma were investigated in all patients examined during both periods (Supplementary Table 2). Compared with cutaneous neurofibroma, for plexiform neurofibroma, pain was significantly more common as the reason to undergo surgery (nodular or diffuse plexiform neurofibroma). In contrast, tumor burden was significantly more common as the reason to undergo surgery for cutaneous neurofibroma than for plexiform neurofibroma.

## Discussion

The present study reported the real-world settings for the surgical treatment of neurofibroma in patients with NF1 with and without restricted medical access.

Regardless of whether medical access was restricted, the main population who underwent surgery for neurofibroma was middle-aged female patients with multiple or severe neurofibromas and mild neurological and bone symptoms. Female patients in their 20s tended to undergo surgery for neurofibroma regardless of restricted medical access, which may be the reason that there are more female than male patients who underwent surgery for neurofibroma even under restricted medical access. These results suggest that female patients have a more intense interest in improving cutaneous manifestations regardless of whether medical access is restricted. On the other hand, male patients, particularly those in their 20s and 30s, may have less time for surgery due to their obligatory social activities even with restrictions due to the pandemic.

Regardless of whether medical access was restricted, male and female patients who were ≥70 years of age rarely underwent surgery for neurofibroma even though the number and size of cutaneous and subcutaneous neurofibromas increases with aging ^(4), (11)^. In contrast to middle-aged patients, elderly patients appear to reluctantly abandon efforts to reduce cutaneous manifestations.

Regardless of whether medical access was restricted, there was an overwhelming majority of cutaneous neurofibroma over plexiform neurofibroma as the target of surgery. This may be attributed to some patients with plexiform neurofibroma abandoning attempts to undergo surgery due to the associated risks of the condition, such as excessive bleeding and permanent neurological damage. Indeed, the number of patients who underwent surgery for plexiform neurofibroma was decreased during the period of restricted medical access with limited hospital admissions. Surgery for diffuse plexiform neurofibroma commonly requires hospitalization due to its associated high risks, whereas cutaneous neurofibroma may be treated at an outpatient clinic, which appears to have been a factor contributing to the increased rate of surgery for cutaneous neurofibroma even with restricted medical access. A retrospective epidemiological analysis of patients with NF1 with a median age of 30 years showed that plexiform and cutaneous neurofibromas were present in 38.2% (176/460) and 68.9% (331/480) of patients, respectively ^(12)^. Therefore, plexiform neurofibroma is not negligible epidemiologically. There are still large unmet needs in the treatment of plexiform neurofibroma, although they will be partially resolved by the development of non-surgical procedures, such as administration with selumetinib.

In the present study, the number of excised neurofibromas in each patient was classified into 1, 2-9, and ≥10 for the following reasons: We performed general anesthesia when we excised ≥10 objects regardless of their sizes, and the type of anesthesia may have influenced the choice of the patients. Therefore, we classified the patients into the patient group who underwent surgery for ≤9 and ≥10 objects in the study. However, we often experienced patients who wished excision of only one neurofibroma under local or general anesthesia. One situation is that a patient suffered from an unpleasant condition caused by one plexiform neurofibroma or one particular cutaneous neurofibroma. Therefore, we classified the patients into the patient group who underwent surgery for one object and that for ≥2 objects in the study.

The number of patients who cited cosmetic disturbances as the reason to undergo surgery significantly decreased during the restricted medical access period. Concerns regarding their cosmetic appearance may have been reduced by the wearing of face masks and some of these patients appeared to abandon surgery due to restricted medical access. Similarly, the number of patients who cited pain as the reason to undergo surgery also significantly decreased. These results are consistent with data showing that the rate of surgeries for plexiform neurofibroma, which more frequently involves pain than cutaneous neurofibroma, decreased.

Several limitations need to be addressed. The present study was conducted as a retrospective analysis of medical records. The use of a questionnaire may provide stronger evidence. Moreover, only a small number of patients were examined. A larger number of patients is needed to obtain more accurate data. Furthermore, the study was conducted before selumetinib was launched. Therefore, the effects of selumetinib on pediatric patients with plexiform neurofibroma were not considered. Additionally, epidemiological data regarding malignant peripheral nerve sheath tumors was lacking in the study, although the occurrence of the tumor is a critical concern.

In conclusion, patients with NF1, who were mostly middle-aged females with severe cutaneous manifestations and mild extracutaneous manifestations, requested surgery for neurofibromas regardless of restricted medical access.

## Article Information

### Conflicts of Interest

None

### Author Contributions

M. O. contributed to the data collection and wrote the manuscript.

Y. N. designed and analyzed the data and wrote the manuscript.

A. A. is a supervisor and edited the manuscript.

All authors reviewed and approved the final manuscript.

### Approval by Institutional Review Board (IRB)

This study protocol was approved by the Ethics Committee of the Jikei University School of Medicine (approval code: 32-073).

### Data Availability Statement

All raw data are available in Supplementary Table 1.

## Supplement

Supplementary Table 1

Supplementary Table 2
