# Supplementary material for: Real-world Settings for the Surgical Treatment of Neurofibroma in Patients with Neurofibromatosis Type 1
Source: JMA J. 2024 Feb 5;7(2):205–12. doi: 10.31662/jmaj.2023-0161 (PMC11074565; doi:10.31662/jmaj.2023-0161)
Supplement: Supplementary Table 1 [file 2433-3298-7-2-0205-s001.pdf]

Supplementary Table 1. Raw data of the present study

Fundamental patient data			Sites of excised neurofibroma					Subtype of excised neurofibroma			Reason to undergo surgery				DNB classification			
Patient number	Sex	Age (years)	Head/neck	Trunk	Arm/leg	Hand/foot	Number of excised neurofibromas	Cutaneous neurofibroma	Nodular plexiform neurofibroma	Diffuse plexiform neurofibroma	Aesthetic disturbance	Pain of tumor	Itch of tumor	Burden of tumor	D-classification	N-classification	B-classification	Medical access-restricted circumstances
1	F	26.7	no	no	yes	no	1	yes	no	no	no	yes	no	no	3	0	0	yes
2	F	32.1	yes	no	no	yes	2	yes	no	no	no	no	no	yes	1	0	0	yes
3	F	50.5	no	yes	no	no	2	yes	no	no	no	no	no	yes	2	0	0	yes
4	F	40.0	no	yes	no	no	2	yes	no	no	no	no	no	yes	2	0	0	yes
5	F	42.8	no	yes	no	no	2	yes	no	no	no	no	no	yes	2	0	0	yes
6	F	39.6	yes	yes	no	no	2	yes	no	no	no	no	no	yes	2	0	0	yes
7	F	36.1	no	yes	no	no	3	yes	no	no	no	no	no	yes	2	0	0	yes
8	F	26.7	no	yes	yes	no	3	yes	no	no	no	no	no	yes	2	0	0	yes
9	F	33.8	yes	yes	no	no	3	yes	no	no	no	no	no	yes	2	0	0	yes
10	F	35.9	no	yes	no	no	3	yes	no	no	no	no	no	yes	2	0	0	yes
11	F	42.8	no	yes	yes	no	2	yes	no	no	no	no	no	yes	2	0	0	yes
12	F	48.7	yes	yes	no	no	3	yes	no	no	no	no	no	yes	3	0	0	yes
13	F	49.9	no	no	yes	no	2	yes	no	no	no	no	no	yes	3	0	0	yes
14	F	67.0	no	no	yes	yes	2	yes	no	no	no	no	no	yes	3	0	0	yes
15	F	44.2	no	yes	yes	no	3	yes	no	no	no	no	no	yes	3	0	0	yes
16	F	50.1	no	no	yes	yes	3	yes	no	no	no	no	no	yes	3	0	0	yes
17	F	63.5	no	no	yes	yes	2	yes	no	no	no	no	no	yes	3	0	0	yes
18	F	29.8	no	yes	no	no	2	yes	no	no	no	no	no	yes	3	0	0	yes
19	F	41.8	yes	yes	no	no	3	yes	no	no	no	no	no	yes	3	0	0	yes
20	F	58.9	no	no	yes	no	2	yes	no	no	no	no	no	yes	3	0	0	yes
21	F	28.3	yes	no	no	no	1	yes	no	no	no	no	no	yes	3	0	0	yes
22	F	71.8	no	yes	yes	no	2	yes	no	no	no	no	no	yes	3	0	0	yes
23	F	67.8	no	no	yes	yes	2	yes	no	no	no	no	no	yes	3	1	0	yes
24	F	67.1	yes	yes	no	no	2	yes	no	no	no	no	no	yes	3	1	0	yes
25	F	29.2	no	yes	no	no	2	yes	no	no	no	no	no	yes	2	0	1	yes
26	F	33.2	yes	yes	no	no	2	yes	no	no	no	no	no	yes	2	0	1	yes
27	F	21.7	yes	yes	yes	no	3	yes	no	no	no	no	no	yes	2	0	1	yes
28	F	29.7	no	yes	no	no	2	yes	no	no	no	no	no	yes	2	0	1	yes
29	F	60.6	yes	no	no	no	1	yes	no	no	no	no	no	yes	2	0	1	yes
30	F	29.6	no	yes	yes	no	2	yes	yes	no	no	no	no	yes	2	0	1	yes
31	F	69.6	no	yes	yes	no	3	yes	no	no	no	no	no	yes	3	0	1	yes
32	F	41.4	no	yes	no	no	2	yes	no	no	no	no	no	yes	1	1	1	yes
33	F	32.5	no	no	yes	no	3	yes	no	no	no	no	no	yes	2	1	1	yes
34	F	54.1	no	yes	yes	no	3	yes	no	no	no	no	no	yes	2	1	1	yes
35	F	41.2	yes	yes	yes	no	2	yes	no	no	no	no	no	yes	2	1	1	yes
36	F	40.3	no	yes	no	no	2	yes	no	no	no	no	no	yes	2	2	1	yes
37	F	36.0	no	yes	no	no	2	yes	no	no	no	no	no	yes	2	2	1	yes
38	F	53.5	no	yes	no	no	2	yes	no	no	no	no	no	yes	4	1	2	yes
39	F	53.3	no	yes	no	no	3	yes	no	yes	no	no	no	yes	4	1	2	yes
40	F	32.7	no	yes	no	no	2	yes	no	no	no	no	no	yes	2	0	nd	yes
41	F	28.0	no	yes	yes	no	2	yes	no	no	yes	no	no	yes	2	0	0	yes
42	F	67.5	yes	no	yes	no	2	yes	no	no	yes	no	no	yes	4	0	0	yes
43	F	51.3	no	no	yes	yes	2	yes	no	no	no	yes	no	yes	2	0	0	yes
44	F	27.9	yes	no	no	no	2	yes	no	no	no	yes	no	yes	3	0	0	yes
45	F	37.4	no	yes	no	yes	2	yes	no	no	no	yes	no	yes	2	1	1	yes
46	F	43.5	no	no	no	yes	2	yes	no	no	no	yes	no	yes	4	1	1	yes
47	M	8.7	no	yes	no	no	nd	no	yes	yes	no	yes	yes	no	1	1	0	yes
48	M	50.9	no	yes	no	no	2	yes	no	no	no	no	no	yes	2	0	0	yes
49	M	39.7	yes	no	no	no	2	yes	no	no	no	no	no	yes	2	0	0	yes
50	M	55.8	no	yes	no	no	2	yes	no	no	no	no	no	yes	2	0	0	yes
51	M	55.6	no	yes	no	no	2	yes	no	no	no	no	no	yes	2	0	0	yes
52	M	34.2	no	yes	no	no	1	yes	no	no	no	no	no	yes	2	0	0	yes
53	M	59.0	yes	yes	no	no	2	yes	no	no	no	no	no	yes	2	0	0	yes
54	M	55.3	no	yes	no	no	2	yes	no	no	no	no	no	yes	2	0	0	yes
55	M	60.2	no	yes	no	no	3	yes	no	no	no	no	no	yes	2	0	0	yes
56	M	44.0	no	yes	yes	no	3	yes	no	no	no	no	no	yes	2	0	0	yes
57	M	33.8	no	yes	no	no	2	yes	no	no	no	no	no	yes	2	0	0	yes
58	M	44.3	no	yes	no	no	2	yes	no	no	no	no	no	yes	2	0	0	yes
59	M	34.1	no	yes	no	no	2	yes	no	no	no	no	no	yes	2	0	0	yes
60	M	56.0	no	no	yes	no	2	yes	no	no	no	no	no	yes	3	0	0	yes
61	M	56.6	yes	no	no	no	2	yes	no	no	no	no	no	yes	3	0	0	yes
62	M	44.4	yes	yes	no	no	2	yes	yes	no	no	no	no	yes	3	0	0	yes
63	M	60.8	no	yes	no	no	2	yes	no	no	no	no	no	yes	3	1	0	yes
64	M	49.3	yes	no	no	yes	2	yes	no	no	no	no	no	yes	3	1	0	yes
65	M	41.9	no	yes	no	no	2	yes	no	no	no	no	no	yes	2	0	1	yes
66	M	49.2	no	yes	no	no	3	yes	no	no	no	no	no	yes	2	0	1	yes
67	M	48.0	no	yes	yes	no	2	yes	no	no	no	no	no	yes	3	0	1	yes
68	M	73.7	no	yes	no	no	2	yes	no	no	no	no	no	yes	4	1	1	yes
69	M	44.4	no	yes	yes	yes	nd	yes	no	no	no	no	no	yes	4	1	2	yes
70	M	67.9	no	yes	no	no	2	yes	no	no	yes	no	no	yes	2	0	0	yes
71	M	39.4	yes	no	no	no	1	yes	no	no	yes	no	no	yes	2	0	0	yes
72	M	49.5	yes	no	no	no	2	yes	no	no	no	yes	no	yes	4	0	1	yes
73	F	52.3	yes	no	no	no	3	yes	no	no	yes	no	no	no	4	1	2	no
74	F	37.5	no	no	yes	no	2	yes	no	no	no	no	no	yes	2	0	1	no
75	M	20.9	yes	no	no	no	3	yes	no	no	no	no	no	yes	2	0	0	no

76	M	33.5	yes	no	no	no	3	yes	no	no	yes	no	no	no	3	1	0	no
77	M	58.6	no	yes	yes	no	3	yes	no	no	no	no	no	yes	4	1	1	no
78	F	48.5	no	yes	yes	no	2	yes	no	no	no	no	no	yes	2	0	0	no
79	M	48.1	no	yes	no	no	2	yes	no	no	no	no	no	yes	3	0	1	no
80	M	21.5	no	yes	no	no	1	no	yes	no	no	yes	no	no	3	0	1	no
81	M	26.9	yes	no	no	no	2	yes	no	no	no	no	no	yes	3	0	0	no
82	F	40.0	yes	no	no	no	2	yes	no	no	yes	no	no	no	3	2	1	no
83	M	18.3	no	yes	no	no	1	no	no	yes	no	no	no	yes	2	0	1	no
84	F	29.5	no	yes	no	no	2	yes	no	no	no	no	no	yes	2	0	0	no
85	M	47.8	yes	yes	yes	no	3	yes	no	yes	no	yes	no	yes	3	0	2	no
86	F	71.1	yes	no	no	no	2	yes	no	no	yes	no	no	no	4	1	2	no
87	F	41.2	yes	no	no	no	2	yes	no	no	no	no	no	yes	2	0	1	no
88	F	46.9	no	yes	yes	no	2	yes	no	no	no	no	no	yes	3	0	0	no
89	M	45.4	no	yes	no	no	2	yes	no	no	no	no	no	yes	2	0	1	no
90	M	68.6	no	yes	no	no	3	yes	no	no	no	no	no	yes	3	1	0	no
91	M	36.8	no	no	yes	no	2	yes	no	no	no	no	no	yes	2	0	0	no
92	F	15.7	no	no	yes	no	1	no	no	yes	no	no	no	yes	3	0	0	no
93	M	44.2	yes	no	no	no	2	yes	no	no	yes	no	no	no	4	1	0	no
94	M	54.5	yes	no	no	no	2	yes	no	no	no	no	no	yes	3	0	1	yes
95	M	54.6	no	no	yes	no	2	yes	no	no	no	no	no	yes	3	0	0	yes
96	F	19.9	yes	no	no	no	1	no	yes	no	yes	no	no	no	3	0	1	yes
97	F	47.2	no	yes	no	no	2	yes	no	no	no	no	no	yes	3	0	0	yes
98	F	45.2	no	yes	no	no	1	yes	no	no	no	no	no	yes	1	0	0	yes
99	F	29.9	no	yes	no	no	2	yes	no	no	no	no	no	yes	2	0	0	yes
100	M	32.9	no	no	yes	no	2	yes	no	no	no	yes	no	no	4	1	1	yes
101	F	26.6	no	yes	no	yes	2	yes	yes	no	no	yes	no	no	4	1	0	yes
102	F	53.6	no	yes	yes	no	2	yes	no	no	no	no	no	yes	4	0	0	yes
103	F	35.0	no	yes	yes	no	2	yes	no	no	no	no	no	yes	2	2	1	yes
104	F	61.3	yes	no	yes	no	2	yes	no	no	no	yes	no	no	4	1	1	yes
105	M	21.5	no	no	yes	no	2	yes	no	no	no	yes	no	no	2	0	0	yes
106	F	58.0	no	yes	yes	no	3	yes	no	no	no	no	no	yes	3	1	0	yes
107	F	42.4	no	no	no	yes	1	yes	no	no	no	yes	no	yes	4	1	1	yes
108	M	20.1	no	no	no	yes	1	yes	no	no	no	no	no	yes	2	0	0	yes
109	M	18.2	yes	no	no	no	1	yes	no	no	no	yes	no	no	1	0	0	yes
110	M	32.1	no	yes	yes	no	3	yes	no	no	no	no	no	yes	2	0	0	yes
111	F	26.4	yes	no	yes	no	2	yes	no	no	no	no	no	yes	3	0	0	yes
112	F	59.1	no	no	yes	no	1	no	no	yes	no	yes	no	yes	4	1	2	yes
113	F	43.2	yes	no	no	no	3	yes	no	no	yes	no	no	no	3	0	0	yes
114	M	51.1	no	yes	no	no	2	yes	no	no	no	no	no	yes	3	0	0	yes
115	M	26.6	no	yes	no	no	3	yes	no	no	no	no	no	yes	4	0	0	yes
116	F	47.5	yes	no	yes	yes	2	yes	no	no	no	yes	no	yes	2	0	0	yes
117	M	33.1	no	no	no	yes	2	yes	no	no	no	no	no	yes	4	1	1	yes
118	M	71.3	no	yes	no	no	3	yes	no	no	no	no	no	yes	3	1	1	yes
119	F	49.7	yes	no	no	no	2	yes	no	no	yes	no	no	no	4	1	2	yes
120	F	66.6	yes	no	no	yes	2	yes	no	no	no	no	no	yes	3	1	0	yes
121	F	50.7	yes	no	no	no	2	yes	no	no	no	no	no	yes	3	1	1	yes
122	F	30.8	yes	no	no	yes	2	yes	no	no	no	no	no	yes	2	1	2	yes
123	F	39.5	no	no	yes	no	2	yes	no	no	no	no	no	yes	2	0	0	yes
124	F	75.7	yes	no	no	no	1	yes	no	no	no	no	no	yes	2	0	0	yes
125	M	21.7	no	yes	yes	no	3	yes	no	no	no	no	no	yes	2	0	0	yes
126	M	32.0	yes	no	no	no	1	yes	no	no	no	no	no	yes	2	0	0	yes
127	F	47.6	no	yes	no	no	2	yes	no	no	no	no	no	yes	4	0	0	yes
128	M	44.2	yes	no	yes	no	2	yes	no	no	no	no	no	yes	3	0	0	yes
129	F	43.2	no	yes	no	no	3	yes	no	no	no	no	no	yes	4	1	1	yes
130	M	32.4	no	no	yes	no	2	yes	no	no	no	no	no	yes	3	0	0	yes
131	F	28.1	yes	no	yes	no	2	yes	no	no	no	no	no	yes	4	1	0	yes
132	M	27.6	yes	no	no	no	1	yes	no	no	yes	no	no	no	2	0	0	yes
133	M	69.3	yes	yes	no	no	3	yes	no	no	no	no	no	yes	3	1	0	yes
134	M	55.2	yes	yes	yes	yes	1	yes	no	no	no	no	no	yes	3	0	1	yes
135	M	51.2	no	yes	no	no	2	yes	no	no	no	no	no	yes	2	0	0	yes
136	M	21.8	no	yes	no	no	2	yes	no	no	no	no	no	yes	2	0	0	yes
137	F	34.6	yes	no	no	no	1	yes	no	no	no	no	no	yes	2	0	0	yes
138	M	67.9	no	yes	no	no	3	yes	no	no	no	no	no	yes	4	1	2	yes
139	M	39.4	no	yes	no	no	3	yes	no	no	no	no	no	yes	4	1	1	yes
140	M	43.9	no	yes	yes	no	2	yes	no	no	no	no	no	yes	2	0	0	yes
141	F	37.9	yes	no	yes	yes	2	yes	no	no	no	no	no	yes	2	0	1	yes
142	F	37.4	yes	no	no	yes	2	yes	no	no	no	no	no	yes	3	1	1	yes
143	M	16.6	no	yes	no	no	2	yes	no	no	no	no	no	yes	2	0	0	yes
144	F	34.5	no	yes	yes	yes	2	yes	no	no	no	no	no	yes	4	1	0	yes
145	F	67.7	yes	yes	no	no	2	yes	no	no	yes	no	no	yes	3	0	0	no
146	M	67.5	no	yes	no	no	2	yes	no	no	no	no	no	yes	3	1	0	no
147	M	46.7	no	yes	no	no	1	no	yes	no	no	no	no	yes	2	0	1	no
148	F	36.6	no	no	yes	no	1	yes	no	no	no	no	no	yes	3	1	1	no
149	F	46.5	no	yes	yes	no	2	yes	no	no	no	yes	no	no	3	0	0	no
150	F	36.8	yes	no	no	no	2	yes	no	no	no	no	no	yes	3	0	0	no
151	F	54.7	no	no	yes	no	1	no	no	yes	no	yes	no	no	4	0	0	no
152	M	42.5	yes	yes	no	no	2	yes	no	no	yes	no	no	yes	3	0	1	no
153	F	30.6	no	yes	no	no	3	yes	no	no	no	no	no	yes	4	0	2	no
154	M	20.9	no	yes	no	no	1	no	no	yes	no	no	no	yes	4	0	0	no
155	M	54.7	yes	no	no	no	2	yes	no	no	yes	no	no	yes	3	2	0	no
156	F	50.6	yes	no	no	no	2	yes	no	no	no	no	no	yes	4	1	2	no
157	M	44.3	no	yes	no	yes	3	yes	no	no	no	yes	no	yes	3	0	0	no
158	F	62.9	no	yes	no	no	2	yes	no	no	no	yes	no	no	3	0	0	no
159	M	37.3	yes	no	no	no	3	yes	no	no	no	no	no	yes	4	0	0	no
160	F	53.8	no	yes	no	no	2	yes	no	no	no	no	no	yes	2	1	0	no
161	F	48.6	yes	no	no	no	2	yes	no	no	no	no	no	yes	3	0	0	no

162	M	41.7	no	yes	no	no	2	yes	no	no	no	no	no	yes	4	1	2	no
163	F	45.9	yes	no	yes	no	2	yes	no	no	no	no	no	yes	3	0	0	no
164	M	47.1	no	yes	no	no	2	yes	no	no	no	no	no	yes	3	0	1	no
165	M	15.2	no	yes	no	no	1	no	yes	no	no	no	no	yes	1	0	0	no
166	M	65.2	no	no	yes	no	2	yes	no	no	yes	no	no	no	3	0	0	no
167	F	30.9	no	yes	no	no	3	yes	no	no	no	no	no	yes	2	1	2	no
168	M	69.8	no	no	yes	no	2	yes	no	no	no	yes	no	yes	3	1	1	no
169	F	41.6	no	yes	no	no	3	yes	no	no	no	no	no	yes	4	0	0	no
170	F	69.6	no	yes	yes	no	3	yes	no	no	no	no	no	yes	3	1	0	no
171	M	38.4	yes	yes	no	no	2	yes	no	no	no	no	no	yes	3	0	0	no
172	M	44.5	no	no	yes	no	2	yes	no	no	no	no	no	yes	3	0	1	no
173	F	51.9	no	yes	no	no	3	yes	no	no	no	no	no	yes	4	0	2	no
174	M	22.5	no	no	yes	no	1	no	yes	yes	no	no	no	yes	4	1	0	no
175	F	43.7	yes	yes	no	yes	3	yes	no	yes	yes	no	no	yes	3	0	0	no
176	F	49.2	yes	no	no	no	2	yes	no	no	yes	no	no	no	3	1	1	no
177	M	57.7	no	yes	no	no	3	yes	no	no	no	no	no	yes	4	1	1	no
178	F	50.4	yes	no	yes	no	2	yes	no	no	no	no	no	yes	4	1	0	no
179	F	47.6	no	yes	no	no	2	yes	no	no	no	no	no	yes	3	0	1	no
180	M	35.7	yes	yes	no	yes	2	yes	no	no	no	no	no	yes	3	0	0	no
181	M	49.6	yes	no	yes	no	2	yes	no	no	yes	no	no	no	3	0	0	no
182	F	52.3	no	no	yes	no	1	yes	no	no	no	no	no	yes	4	2	1	no
183	M	47.4	no	yes	no	no	2	yes	no	no	no	no	no	yes	3	0	1	no
184	M	20.2	yes	no	no	no	2	yes	no	no	no	no	no	yes	2	0	0	no
185	F	64.5	no	yes	yes	no	3	yes	no	no	no	no	no	yes	3	0	0	no
186	M	38.8	no	yes	no	no	2	yes	no	no	no	no	no	yes	4	0	0	no
187	F	34.9	no	yes	yes	no	2	yes	no	no	no	yes	yes	yes	4	1	0	no
188	M	43.5	yes	no	no	no	2	yes	no	no	no	no	no	yes	3	0	0	no
189	M	49.6	no	yes	no	no	1	yes	no	no	no	no	no	yes	3	1	0	no
190	F	38.1	no	no	yes	no	2	yes	no	no	no	no	no	yes	3	0	0	no
191	M	70.1	yes	yes	yes	no	2	yes	no	no	no	yes	no	yes	3	0	1	no
192	F	40.7	yes	yes	no	no	2	yes	no	no	no	no	no	yes	3	0	0	no
193	M	39.1	no	no	yes	no	1	yes	no	no	no	no	no	yes	2	0	0	no
194	F	68.1	yes	no	no	no	2	yes	no	no	yes	no	no	yes	3	0	0	no
195	F	33.8	no	yes	yes	no	2	yes	no	no	no	no	no	yes	4	1	1	no
196	M	46.2	yes	yes	yes	no	2	yes	no	no	no	no	no	yes	3	0	0	no
197	M	42.0	yes	no	yes	no	2	yes	no	no	no	no	no	yes	4	1	2	no
198	M	76.4	no	no	no	yes	2	yes	no	no	yes	no	no	yes	4	0	0	no
199	F	26.4	yes	yes	no	no	2	yes	no	no	no	no	no	yes	3	1	0	no
200	M	58.7	no	yes	no	no	2	yes	no	no	no	no	no	yes	4	1	0	no
201	F	30.7	yes	yes	no	no	2	yes	no	no	no	no	no	yes	3	1	0	no
202	F	48.5	yes	no	no	no	2	yes	no	no	yes	no	no	no	4	1	1	no
203	M	54.5	yes	no	yes	no	2	yes	no	no	no	no	no	yes	3	0	0	no
204	F	52.6	no	no	yes	no	1	yes	no	no	no	no	no	yes	4	2	0	no
205	M	58.2	no	yes	yes	no	3	yes	no	no	no	no	no	yes	4	1	1	no
206	M	65.6	no	no	yes	no	2	yes	no	no	no	no	no	yes	3	0	0	no
207	F	63.2	no	no	yes	yes	2	yes	yes	no	no	yes	no	yes	4	0	0	no
208	F	46.3	no	no	yes	no	2	yes	no	no	no	no	no	yes	3	0	0	no
209	M	44.6	no	yes	no	no	2	yes	no	no	no	no	no	yes	3	0	0	no
210	M	12.6	no	no	yes	no	1	no	no	yes	yes	no	no	yes	4	0	0	no
211	F	32.3	yes	yes	no	no	2	yes	no	no	no	no	no	yes	2	0	0	no
212	F	34.0	no	yes	yes	no	2	yes	no	no	no	no	no	yes	2	2	1	no
213	M	47.5	yes	yes	no	no	2	yes	no	no	no	no	no	yes	3	0	1	no
214	F	37.0	no	yes	no	no	1	no	yes	no	no	no	no	yes	3	1	1	no
215	F	46.1	no	no	yes	no	1	no	no	yes	no	yes	no	no	3	2	1	no
216	F	37.5	no	yes	yes	no	2	yes	no	yes	no	no	no	yes	4	0	0	no
217	M	36.2	no	yes	no	no	2	yes	no	no	no	no	no	yes	3	0	0	no
218	M	44.9	no	yes	no	no	3	yes	no	no	no	no	no	yes	3	0	1	no
219	F	37.3	yes	no	no	yes	2	yes	no	no	no	no	no	yes	4	0	0	no
220	F	77.6	no	yes	no	no	3	yes	no	no	no	no	no	yes	3	0	1	no
221	M	68.1	no	no	yes	no	1	no	yes	no	no	yes	no	no	3	1	0	no
222	M	20.5	yes	no	no	no	2	yes	no	no	no	no	no	yes	2	0	0	no
223	M	42.2	yes	yes	yes	no	2	yes	no	no	no	no	no	yes	4	1	2	no
224	M	33.6	yes	no	no	no	1	yes	no	no	yes	no	no	no	2	0	0	no
225	F	49.7	no	no	yes	yes	3	yes	no	no	yes	no	no	yes	2	1	1	no
226	M	70.1	yes	no	no	no	1	yes	no	no	yes	no	no	yes	4	0	1	no
227	F	48.2	yes	no	no	no	1	yes	no	no	yes	no	no	yes	4	1	0	no
228	M	50.0	no	no	yes	no	2	yes	no	no	no	no	no	yes	3	0	0	no
229	F	47.2	no	no	yes	no	2	yes	no	no	no	no	no	yes	3	0	0	no
230	F	60.3	no	yes	no	no	3	yes	no	no	no	no	no	yes	2	0	0	no
231	F	66.2	yes	yes	yes	no	3	yes	no	no	no	no	no	yes	3	0	2	no
232	M	47.9	no	no	yes	no	2	yes	no	no	no	no	no	yes	3	0	1	no
233	F	55.2	no	no	yes	no	1	no	no	yes	no	yes	no	yes	3	0	0	no
234	F	19.2	yes	no	no	no	2	yes	no	no	yes	no	no	no	2	1	2	no
235	M	57.0	yes	no	no	yes	2	yes	no	no	yes	no	no	no	4	1	0	no
236	M	45.5	no	no	yes	yes	2	yes	no	no	no	no	no	yes	3	0	0	no
237	M	48.0	no	no	yes	no	1	no	no	yes	no	yes	no	yes	3	1	0	no
238	F	82.4	no	yes	no	no	1	no	yes	no	no	yes	no	no	4	0	0	no
239	F	38.5	no	no	yes	no	2	yes	no	no	no	no	no	yes	3	0	0	no
240	F	52.4	no	yes	no	no	3	yes	no	no	no	no	no	yes	4	0	2	no
241	M	70.2	no	no	yes	no	3	yes	no	no	no	yes	no	yes	3	1	1	no
242	M	20.1	no	yes	no	no	2	yes	no	no	no	yes	no	yes	3	0	0	no
243	F	38.4	no	yes	no	no	2	yes	no	no	no	no	no	yes	3	1	0	no
244	M	36.4	yes	no	yes	no	2	yes	no	no	no	no	no	yes	3	0	0	no
245	F	64.5	no	no	no	yes	2	yes	no	no	no	yes	no	yes	3	0	0	no
246	F	52.4	yes	no	no	no	2	yes	no	no	yes	no	no	no	3	1	0	no
247	M	32.2	no	yes	no	no	1	no	no	yes	no	no	no	yes	4	1	1	no

248	F	29.2	yes	yes	yes	no	2	yes	no	no	no	no	no	yes	2	0	0	no
249	M	54.1	no	no	yes	no	2	yes	no	no	no	no	no	yes	3	0	0	no
250	F	36.3	yes	no	no	no	2	yes	no	no	no	no	no	yes	2	2	1	no
251	M	45.0	no	no	yes	no	2	yes	no	no	no	no	no	yes	3	0	0	no
252	F	68.6	no	no	yes	yes	2	yes	no	no	no	no	no	yes	3	0	0	no
253	F	42.3	no	yes	yes	no	3	yes	no	no	no	no	no	yes	4	0	0	no
254	F	42.7	no	no	no	yes	1	yes	no	no	no	yes	no	yes	2	0	0	no
255	F	46.6	no	yes	yes	no	2	yes	no	no	no	no	no	yes	3	0	0	no
256	M	45.1	no	yes	no	no	3	yes	no	no	no	no	no	yes	3	0	0	no
257	F	41.3	no	yes	no	no	2	yes	no	no	no	no	no	yes	3	0	0	no
258	F	57.8	no	yes	no	no	2	yes	no	no	no	no	no	yes	2	0	0	no
259	M	57.5	no	yes	no	no	3	yes	no	no	no	no	no	yes	3	1	0	no
260	M	65.9	no	no	yes	no	2	yes	no	no	yes	no	no	no	3	0	0	no
261	F	48.2	no	yes	no	no	1	yes	no	no	no	no	no	yes	3	0	1	no
262	F	57.4	yes	no	no	no	2	yes	no	no	yes	no	no	no	3	0	1	no
263	F	37.7	no	yes	yes	no	3	yes	no	no	no	no	no	yes	3	0	0	no
264	F	28.7	no	yes	yes	no	2	yes	no	no	no	no	no	yes	4	0	0	no
265	F	51.5	yes	no	no	no	2	yes	no	no	yes	no	no	yes	4	1	2	no
266	M	44.1	no	yes	no	no	2	yes	no	no	no	no	no	yes	2	0	0	no
267	F	33.8	yes	yes	yes	no	2	yes	no	no	no	no	no	yes	4	0	0	no
268	F	49.8	no	no	yes	no	3	yes	no	no	no	no	no	yes	2	0	0	no
269	F	30.8	no	yes	no	no	2	yes	no	no	no	no	no	yes	3	1	2	no
270	M	50.3	yes	no	no	no	1	no	yes	no	yes	no	no	no	3	0	0	no
271	M	46.2	yes	no	yes	no	2	yes	no	no	no	yes	no	yes	3	1	0	no
272	M	64.1	yes	no	no	no	2	yes	no	no	yes	no	no	no	2	0	0	no
273	F	60.4	yes	no	no	no	2	yes	no	no	yes	no	no	no	2	0	1	no
274	F	47.3	yes	yes	no	no	2	yes	no	no	no	no	no	yes	3	0	0	no
275	F	54.2	no	yes	no	no	3	yes	no	no	no	no	no	yes	3	0	0	no
276	M	63.3	no	yes	yes	no	3	yes	no	no	no	no	no	yes	3	0	0	no
277	M	61.0	yes	no	no	no	3	yes	no	no	yes	no	no	no	3	0	0	no
278	F	60.6	no	yes	no	no	3	yes	no	no	no	no	no	yes	3	1	1	no
279	F	40.4	yes	no	no	no	2	yes	no	no	yes	no	no	no	4	1	1	no
280	F	35.8	yes	yes	yes	no	3	yes	no	no	no	no	no	yes	2	0	0	no
281	F	49.6	yes	yes	no	no	2	yes	no	no	no	no	no	yes	4	1	2	no
282	M	37.4	no	yes	yes	no	2	yes	no	no	no	no	no	yes	3	1	0	no
283	M	47.9	no	yes	no	no	3	yes	no	no	no	no	no	yes	2	0	0	no
284	F	56.4	yes	no	no	no	1	no	no	yes	no	yes	no	no	4	1	2	no
285	F	22.6	no	yes	yes	no	2	yes	no	no	no	no	no	yes	2	1	2	no
286	M	45.7	no	no	yes	no	2	yes	no	no	no	no	no	yes	4	0	1	no
287	M	19.0	yes	yes	no	no	2	no	no	yes	no	yes	no	yes	3	1	0	no
288	F	35.7	no	yes	yes	no	3	yes	no	no	no	no	no	yes	3	1	1	no
289	M	46.1	yes	no	yes	no	2	yes	no	no	yes	no	no	yes	3	0	1	no
290	M	40.7	no	yes	yes	no	2	yes	no	no	no	no	no	yes	4	1	2	no
291	M	56.8	no	yes	no	no	3	yes	no	no	no	no	no	yes	3	0	0	no
292	M	61.9	no	yes	no	no	3	yes	no	no	no	no	no	yes	3	0	0	no
293	F	40.6	yes	yes	yes	no	3	yes	no	no	yes	no	no	yes	3	0	0	no
294	F	24.2	yes	yes	no	no	2	yes	no	no	no	no	no	yes	4	0	0	no
295	M	49.8	no	yes	no	no	1	no	no	yes	no	yes	no	yes	4	0	2	no
296	F	35.9	yes	yes	no	no	2	yes	no	no	yes	no	no	yes	2	0	0	no
297	F	56.9	no	no	yes	no	2	yes	no	no	no	no	no	yes	2	0	0	no
298	M	41.2	no	yes	no	no	2	yes	no	no	no	no	no	yes	2	0	0	no
299	M	43.5	no	yes	yes	no	2	yes	no	no	no	no	no	yes	2	0	1	no
300	F	66.2	yes	yes	no	no	3	yes	no	no	no	yes	no	yes	3	0	1	no
301	F	23.9	yes	no	no	no	1	no	no	yes	no	yes	no	no	2	1	1	no
302	F	75.9	no	no	no	yes	1	no	yes	no	no	yes	no	no	4	1	0	no
303	M	31.9	no	yes	no	no	2	no	yes	no	no	yes	no	no	4	2	1	no
304	F	60.9	no	yes	yes	no	3	yes	no	no	no	no	no	yes	3	1	1	no
305	F	25.7	no	yes	no	no	1	no	no	yes	no	yes	no	yes	2	0	0	no
306	F	37.0	no	no	no	yes	2	yes	no	no	no	yes	no	yes	2	0	0	no
307	M	46.2	yes	no	no	no	2	yes	no	no	yes	no	no	no	3	0	1	no
308	F	55.8	no	yes	no	no	2	yes	no	no	yes	no	no	no	2	0	0	no
309	M	75.3	yes	no	yes	no	2	yes	no	no	no	no	no	yes	4	0	0	no
310	F	27.1	yes	yes	no	no	2	yes	no	no	no	no	no	yes	3	0	0	no
311	F	39.7	no	yes	no	no	2	yes	no	no	no	yes	no	no	2	0	0	no
312	F	51.0	no	yes	no	no	3	yes	no	no	no	no	no	yes	2	1	1	no
313	M	48.6	no	yes	no	no	2	yes	no	no	no	no	no	yes	2	0	0	no
314	F	44.7	no	no	no	yes	2	yes	no	no	yes	no	yes	yes	3	0	1	no
315	F	68.8	no	yes	no	yes	2	yes	no	yes	no	yes	no	yes	3	1	0	no
316	F	32.9	yes	yes	yes	no	2	yes	no	no	yes	yes	no	yes	4	0	1	no
317	F	63.5	yes	no	no	no	3	yes	no	no	yes	no	no	yes	3	0	0	no
318	F	35.9	no	no	no	yes	1	yes	no	no	no	yes	no	yes	3	1	1	no
319	F	67.1	yes	no	yes	no	2	yes	no	no	yes	no	no	no	3	0	0	no
320	M	41.6	yes	yes	yes	no	2	yes	no	no	yes	no	no	yes	2	0	0	no
321	F	40.9	yes	yes	no	no	2	yes	yes	no	no	yes	no	yes	3	0	0	no
322	F	37.3	no	yes	yes	yes	nd	yes	no	no	no	yes	no	yes	3	1	0	no
323	M	62.1	no	yes	no	no	2	yes	no	no	no	no	no	yes	3	0	0	no
324	F	45.1	no	yes	no	yes	2	yes	no	no	yes	yes	no	no	3	0	0	no
325	M	64.5	yes	no	no	no	2	yes	no	no	yes	no	no	no	2	0	0	no
326	F	33.0	no	yes	yes	no	2	yes	no	no	no	no	no	yes	4	0	1	no
327	F	76.0	yes	no	no	yes	2	yes	yes	no	no	yes	no	yes	4	1	0	no
328	F	36.1	yes	yes	no	no	3	yes	no	no	no	no	no	yes	2	0	0	no
329	M	53.4	no	yes	no	no	2	yes	no	no	no	no	no	yes	3	0	0	no
330	M	37.9	no	yes	no	no	2	yes	no	no	no	no	no	yes	4	0	1	no
331	F	57.2	yes	no	no	no	3	yes	no	no	yes	no	no	yes	2	0	0	no
332	M	69.0	no	no	no	no	3	yes	no	no	no	yes	no	yes	3	1	1	no
333	F	41.0	yes	no	no	yes	1	yes	no	no	no	no	no	yes	3	0	0	no

334	F	46.0	no	yes	no	no	2	yes	no	no	no	no	no	yes	2	0	0	no
335	M	43.7	yes	yes	no	yes	3	yes	no	no	no	no	no	yes	3	0	0	no
336	M	57.8	no	yes	yes	no	2	yes	yes	no	no	yes	no	yes	4	1	0	no
337	M	42.7	no	no	yes	no	2	yes	no	no	no	yes	no	yes	3	0	0	no
338	M	67.0	yes	yes	no	no	3	yes	no	no	no	no	no	yes	3	0	0	no
339	M	59.3	no	no	yes	no	1	yes	no	no	no	yes	no	yes	3	0	0	no
340	M	54.2	no	yes	yes	no	3	yes	no	no	no	no	no	yes	4	0	0	no
341	F	41.0	no	yes	yes	no	3	yes	no	no	no	no	no	yes	3	0	0	no
342	M	46.6	no	yes	no	yes	2	yes	no	no	no	no	no	yes	3	0	1	no
343	M	46.8	no	no	yes	yes	2	yes	no	no	no	yes	no	yes	3	1	0	no
344	M	53.7	yes	yes	yes	no	3	yes	no	no	no	yes	no	yes	3	0	0	no
345	F	50.2	yes	yes	no	no	2	yes	no	no	no	no	no	yes	4	1	2	no
346	F	47.9	no	no	no	yes	2	yes	no	no	no	yes	no	yes	3	0	0	no
347	F	56.2	no	yes	yes	no	2	yes	no	no	no	no	no	yes	2	0	0	no
348	F	51.9	no	yes	no	no	3	yes	no	no	no	no	no	yes	4	1	1	no
349	M	41.1	no	yes	no	no	2	yes	no	no	no	no	no	yes	4	1	2	no
350	M	57.3	no	yes	no	no	3	yes	no	no	no	no	no	yes	4	1	1	no
351	F	37.6	no	yes	yes	no	2	yes	no	no	no	no	no	yes	3	1	0	no
352	M	15.7	no	no	yes	no	1	no	yes	no	no	no	no	yes	2	0	1	no
353	M	19.1	no	yes	no	no	2	no	yes	no	no	yes	no	yes	2	0	0	no
354	F	36.4	no	no	no	yes	2	yes	no	no	no	yes	no	yes	2	0	0	no
355	F	50.8	yes	no	no	no	1	yes	no	no	no	no	no	yes	2	0	0	no
356	F	46.0	yes	yes	no	no	2	yes	no	no	no	yes	no	yes	2	0	0	no
357	M	69.3	no	no	yes	yes	2	yes	no	no	no	yes	no	yes	3	1	1	no
358	M	53.7	no	yes	no	no	2	yes	no	no	no	no	no	yes	3	0	0	no
359	F	24.5	no	no	no	yes	2	yes	no	no	no	no	no	yes	2	0	0	no
360	F	72.8	no	yes	no	no	2	yes	no	no	no	yes	no	yes	3	1	2	no
361	F	34.0	yes	no	no	no	2	yes	no	no	no	no	no	yes	3	1	2	no
362	M	64.8	no	yes	no	no	2	yes	no	no	no	no	no	yes	2	0	0	no
363	F	47.8	no	no	yes	no	2	yes	no	no	yes	no	no	yes	4	1	1	no
364	F	50.9	yes	no	no	no	2	yes	no	no	no	yes	no	yes	2	0	0	no
365	F	34.3	no	yes	yes	no	3	yes	no	no	no	no	no	yes	4	1	0	no
366	M	44.1	no	yes	no	no	2	yes	no	no	no	no	no	yes	2	0	0	no
367	F	45.6	no	no	yes	no	2	yes	no	no	no	no	no	yes	3	0	0	no
368	F	51.5	no	yes	no	no	3	yes	yes	no	no	yes	no	yes	2	1	1	no
369	F	55.4	no	yes	no	no	3	yes	yes	no	no	yes	no	yes	4	0	1	no
370	M	41.4	no	yes	yes	no	3	yes	yes	no	no	yes	no	yes	4	1	2	no
371	M	56.5	no	yes	no	no	3	yes	yes	no	no	no	no	yes	3	1	0	no
372	F	61.2	no	yes	no	no	2	yes	no	no	no	no	no	yes	2	0	0	no
373	F	37.6	no	no	yes	no	1	yes	no	no	no	no	no	yes	2	0	0	no
374	M	46.8	no	yes	no	no	1	no	no	yes	no	yes	no	yes	3	0	1	no
375	F	41.9	no	no	yes	no	2	yes	no	no	no	no	no	yes	3	0	0	no
376	M	16.4	no	no	yes	no	1	no	no	yes	no	yes	no	yes	2	0	0	no
377	F	47.9	no	no	yes	yes	2	yes	no	yes	no	yes	no	yes	3	0	0	no
378	M	19.8	yes	yes	no	no	2	yes	no	no	no	no	no	yes	1	0	0	no
379	F	21.6	yes	yes	no	no	2	yes	no	no	yes	no	no	yes	1	0	0	no
380	F	58.3	yes	no	yes	no	2	yes	no	no	no	no	no	yes	2	0	1	no
381	M	43.1	no	yes	no	no	2	yes	no	no	no	no	no	yes	3	0	0	no
382	M	47.1	no	yes	no	no	1	no	no	yes	no	yes	no	yes	3	0	1	no
383	F	18.4	yes	no	no	no	1	no	yes	no	no	yes	no	no	4	1	1	no

nd, no data
