# Supplementary material for: Real-world Settings for the Surgical Treatment of Neurofibroma in Patients with Neurofibromatosis Type 1
Source: JMA J. 2024 Feb 5;7(2):205–12. doi: 10.31662/jmaj.2023-0161 (PMC11074565; doi:10.31662/jmaj.2023-0161)
Supplement: Supplementary Table 2 [file 2433-3298-7-2-0205-s002.pdf]

Supplementary Table 2. Comparison of reasons to undergo surgery between neurofibroma subtypes

Reasons to excise cutaneous NF	Cutaneous (%)	Non-cutaneous (%)	*p*-value
Cosmetic disturbance	51/350 (14.6)	3/33 (9.1)	0.384
Pain of tumor	52/350 (14.9)	21/33 (63.6)	<0.001*
Itch of tumor	2/350 (0.6)	1/33 (0.3)	0.126
Burden of tumor	314/350 (89.7)	20/33 (60.6)	<0.001*

Reasons to excise nodular plexiform NF	Nodular (%)	Non-nodular (%)	*p*-value
Cosmetic disturbance	2/26 (7.7)	52/357 (14.6)	0.331
Pain of tumor	16/26 (61.5)	57/357 (16.0)	<0.001*
Itch of tumor	1/26 (3.8)	2/357 (0.6)	0.067
Burden of tumor	16/26 (61.5)	318/357 (89.1)	<0.001*

Reasons to excise diffuse plexiform NF	Diffuse (%)	Non-diffuse (%)	*p*-value
Cosmetic disturbance	2/26 (7.7)	52/357 (14.6)	0.331
Pain of tumor	17/26 (65.4)	56/357 (15.7)	<0.001*
Itch of tumor	1/26 (3.8)	2/357 (0.6)	0.067
Burden of tumor	21/26 (80.8)	313/357 (87.7)	0.309

NF, neurofibroma. *Statistically significant.
